# Achieving a satisfactory clinical and biochemical response in antiphospholipid syndrome and severe thrombocytopenia with rituximab: two case reports

**DOI:** 10.1002/ccr3.946

**Published:** 2017-04-18

**Authors:** Donia Gamoudi, Melanie Cutajar, Nadia Gamoudi, David James Camilleri, Alex Gatt

**Affiliations:** ^1^ Department of MedicineMater Dei HospitalMsidaMalta; ^2^ Department of Haemato‐OncologyMater Dei HospitalMsidaMalta; ^3^ Department of PathologyFaculty of Medicine and SurgeryUniversity of MaltaMsidaMalta

**Keywords:** Anticoagulation, antiphospholipid syndrome, rituximab, thrombocytopenia, thrombosis

## Abstract

In AP syndrome (APS) with severe thrombocytopenia, rituximab represents a unique drug which can balance the effect of bleeding and thrombosis. By reducing the production of autoantibodies, rituximab can simultaneously raise the platelets and reduce the chance of thrombosis by suppressing APL antibodies. Rituximab can supersede splenectomy as second‐line therapy in similar patients.

Almost 40% of patients presenting with immune thrombocytopenia purpura (ITP) are positive for antiphospholipid antibodies (APAs) 1. The management of similar patients presenting with isolated severe thrombocytopenia associated with APAs is the same as for ITP 2. This includes the use of steroids, intravenous immunoglobulin (IvIg), and second line with splenectomy and immunosuppression. However, ~50% of patients presenting with ITP and incidental APAs will develop clinical thrombosis 1. In fact, there is increased evidence that the heightened thrombotic risk seen in patients with ITP is perhaps related to APAs 3.

We describe two difficult cases with ITP and AP syndrome (APS) and the therapeutic dilemmas in managing their high risk of both bleeding and thrombosis.

## Case 1

A 26‐year‐old lady was referred to Accident & Emergency for investigation of severe thrombocytopenia and resultant menorrhagia. She was otherwise well and denied any family history of bleeding. Her platelets were 1 × 10^9^/L, hemoglobin 10.0 g/dL, MCV 71.1 fL, and normal WBC. Blood picture showed severe thrombocytopenia and signs of iron deficiency confirmed by hypoferritinemia. Renal, liver function, and electrolytes were normal, as was an abdominal ultrasound. APAs were all significantly raised (Table 1), and her lupus anticoagulant by dilute Russell viper venom test (DRVVT) was repeatedly positive. Helicobacter pylori antibodies, hepatitis screen, HIV serology, toxoplasmosis, and an autoimmune screen were all negative. A bone marrow showed depleted iron stores but otherwise normal confirming peripheral platelet consumption and iron deficiency anemia. She was started on oral prednisolone at 1 mg/kg and intravenous immunoglobulins (IVIg) 1 g/kg/day for 2 days. An initial partial response was observed with platelets increasing to 100 × 10^9^/L but 3 weeks later she while still on 1 mg/kg prednisolone presented again with menorrhagia and platelets <10 × 10^9^/L. She received high‐dose steroids (dexamethasone 40 mg daily for 4 days per month) and IVIg, which, however, failed to maintain her platelet count at a level that controlled the bleeding. The patient was intolerant to continued corticosteroid therapy and was started on Azathioprine 50 mg daily, which was increased gradually to 150 mg. Three months later, she relapsed again with epistaxis and platelets of 5 × 10^9^/L. She was again admitted for IV steroids and IVIg administration and was discharged with a platelet count of 120 × 10^9^/L. While steroids were being tailed off, she was admitted with hemorrhagic Varicella Zoster with platelets <10 × 10^9^/L. Azathioprine was discontinued, and she received IV acyclovir, IVIg, and dapsone. Three months later (platelets 88 × 10^9^/L), she presented with sudden onset short‐lived blindness in her right eye. Neurology consult, ophthalmic examination, MRI brain, and angiography were normal. She was diagnosed with amaurosis fugax and started on aspirin. A carotid doppler US was normal, but echocardiography showed a patent foramen ovale. Leg venous Doppler US revealed no clots. We discussed further treatment options with the patient. She was against more steroids or splenectomy. Rituximab at 375 mg/m^2^ once weekly × 4 weeks was opted for. No acute side effects were noted. After a two‐year follow‐up period, she remains well with platelets >150 × 10^9^/L on aspirin. Her APAs have now normalized 40 months post‐rituximab therapy but her lupus anticoagulant remains positive (Table 1).

**Table 1:** Anticardiolipin and antiβ2GP1 antibody levels for Case 1 and Case 2

Date	Anticardiolipin IgG (GPL U/mL)	Anticardiolipin IgM (GPL U/mL)	Antiβ2 GP1 IgG (U/mL)	Antiβ2 GP1 IgM (U/mL)
Case 1				
4‐2010	46.9	8.4	99	66
1‐2012	62.3	18.8	NA	NA
9‐2012	Rituximab			
11‐2012	26.4	5.5	NA	NA
6‐2013	40.3	<5.0	9.6	1.7
12‐2013	39.1	<5.0	>100	3.6
3‐2014	27.0	<5.0	60.7	2.3
3‐2015	18.6	<5.0	23.1	1.1
1‐2016	<8.0	<5.0	3.7	1.2
Case 2				
9‐2011	46.5	5.5	22	<2
1‐2012	24.9	<5.0	NA	NA
12‐2013	88.1	<5.0	NA	NA
3‐2014	45.1	6.7	9.6	1.7
6‐2014	50.7	<5.0	>100	3.6
8‐2014	Rituximab			
10‐2014	83.3	<5.0	60.7	2.3
3‐2015	40	<5.0	23.1	1.1
8‐2015	19.4	<5.0	11.8	<1.0

## Case 2

**Figure 1:**
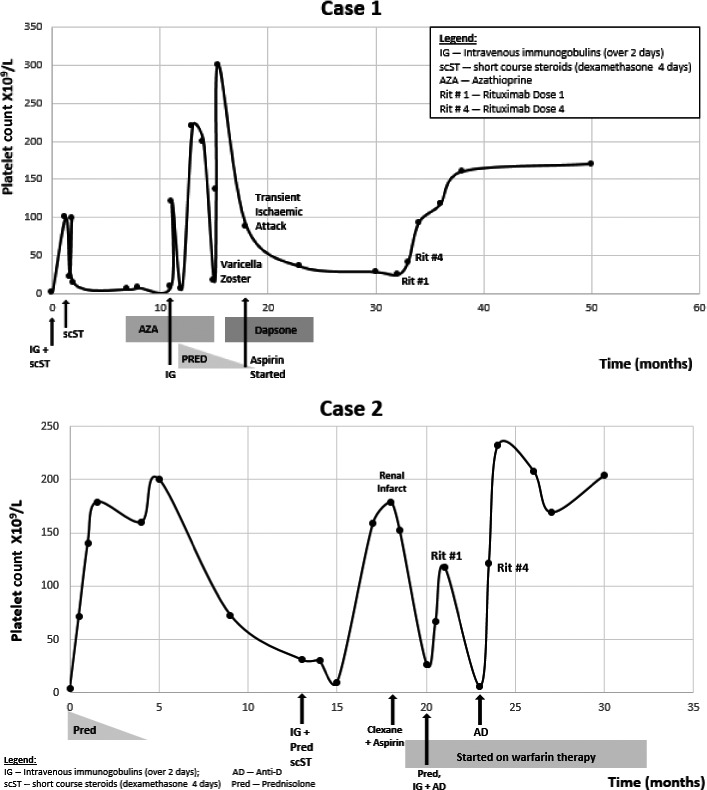
Platelet counts and treatment over time for the two cases. IG intravenous immunoglobulins, scST short‐course steroids, Rit 1 first rituximab dose, Rit 4 fourth rituximab dose, TIA transient ischemic attack, AD Anti‐D.

A 22‐year‐old previously healthy gentleman was referred with easy bruising, mucosal bleeding, and a diffuse purpuric rash. Examination was otherwise unremarkable. Blood tests revealed platelets 4 × 10^9^/L. The same battery of tests as per Case 1 was normal. He had raised ACAs (Table 1) and positive DRVVT. He started prednisolone (1 mg/kg), which was gently tapered and remained well with platelets >100 × 10^9^/L for more than a year. His platelets were then noted to be drifting down (Fig. 1). Initially, he was not administered any treatment as he remained asymptomatic. One month later, he presented with bleeding similar to presentation and platelets of 10 × 10^9^/L. He received 4 days of 40 mg/day dexamethasone and IVIg (2 g/kg). He responded and was discharged with platelets of 220 × 10^9^/L and was kept on monthly high‐dose dexamethasone. Four months later, he was admitted to hospital with sudden onset left‐sided loin pain radiating to the groin (platelets 179 × 10^9^/L). Urinalysis revealed microscopic hematuria, and contrast‐enhanced CT revealed a left renal infarct. He was treated with enoxaparin 1 mg/kg bd and aspirin. ECHO and a leg US venous Doppler were normal. He was later converted to warfarin and remained well with platelets around 25 × 10^9^/L for another 6 weeks, after which he represented with bleeding, a platelet count of 5 × 10^9^/L and an INR of 6.0. He received IVIgs and his steroids were increased without any platelet response. As his blood group was AB positive, he received IV anti‐D (18,000 U), while warfarin anticoagulation was reversed with vitamin K and changed back to enoxaparin with good effect. After discussion with the patient, it was decided to try rituximab (as above). After more than 12‐month follow‐up, he remains well with normal platelets and decreasing antibodies (Table 1). He has since switched back onto warfarin.

These two cases highlight the importance of testing all ITP patients for lupus anticoagulant and antibodies. It also highlights the practical therapeutic difficulties in patients with severe thrombocytopenia and triple‐positive APAs. They both suffered significant thrombotic events after their platelets were “normalized” within 1 year of diagnosis. They both had recurring severe ITP with bleeding while on anticoagulants. Further traditional management options in similar patients with recurring ITP would be either a thrombopoietin (TPO) mimetic or splenectomy to induce more durable remissions 4, 5. We were not happy with using TPO agonists due to lack of experience in similar patients and the possible heightened risk of thrombosis 6. Splenectomy is associated with good short‐ and long‐term platelet responses but has significant drawbacks. Being a major operation, it is not immediately appealing to patients. It is also associated with significant thrombotic morbidity and mortality, which risks might be further augmented in patients with APS 7, 8, 9. Even though rituximab is expensive (local total cost per patient €10,000), splenectomy seems similarly expensive ($16,000) 10. Rituximab seems to be ideal in similar cases as it tackles the condition at its roots by affecting the antibody‐producing cells causing APS and ITP. In fact, in our cases, it achieved an immediate and persistent response without significant side effects. Rituximab has been used in catastrophic APS with good effect 11. Even though ours is a clinical observation, we feel that rituximab should be considered as second‐line therapy in APS and severe thrombocytopenia before splenectomy and TPO mimetics. Similar patients whose yin‐yang has been disturbed by increasing platelet counts should be considered for prophylactic anticoagulation when platelets increase to >50 × 10^9^/L to try to prevent thrombotic events.

## Authorship

DG, MC, NG and AG: wrote the manuscript; DJC and AG: designed the research study; all authors have critically revised the manuscript; and regrettably, NG: has tragically passed away while this manuscript was being peer‐reviewed.

## Conflict of Interest

None declared.
